# Evaluation of an artificial intelligence generated Brock score to determine malignancy risk of screen-detected pulmonary nodules

**DOI:** 10.1016/j.ejro.2026.100765

**Published:** 2026-05-27

**Authors:** Kathryn J. Long, Michael Balassone, Saigopal Sathyamurthy, Vikash Challa, Deepak Adarsh, Gerard A. Silvestri

**Affiliations:** a Cleveland Clinic, 9500 Euclid Ave, Cleveland, OH 44195, United States; b Medical University of South Carolina, 96 Jonathan Lucas St, Charleston, SC 29425, United States; c Qure.ai Technologies, Mumbai, India

**Keywords:** Pulmonary nodule, Lung cancer screening, Artificial Intelligence

## Abstract

**Background:**

Estimation of malignancy risk is a crucial step in the evaluation of indeterminate pulmonary nodules (IPN) on computed tomography (CT) scans. Artificial intelligence (AI) tools can improve the risk prediction of IPN. The purpose of this study was to compare the performance of an AI generated Brock score with standard Brock score to estimate malignancy risk.

**Methods:**

The AI tool qCT (Qure.ai Technologies, Mumbai, India) was tested on a case-control series of patients with screen-detected nodules from the National Lung Cancer Screening Trial (NLST). Standard Brock score and AI Brock score were calculated for each nodule using clinical data from the NLST dataset and nodule characteristics derived from the dataset and AI interpretation of CT images, respectively. Performance of each model was assessed using the area under the receiver operating curve (AUC). The sensitivity and specificity of each model was calculated at fixed thresholds of ≥ 2, 5, and 10% cancer risk.

**Results:**

A total of 478 patients (239 cases, 239 controls) were included. The AUC of the AI Brock score and standard Brock score were 0.72 (95% CI 0.67–0.76) and 0.76 (95% CI 0.72–0.81), respectively (p = 0.053). At a 5% threshold, the sensitivity and specificity were 69% and 65% for AI and 57% and 82% for the standard.

**Conclusion:**

The accuracy of the AI tool was lower than the standard Brock score, though not reaching statistical significance. Across all risk thresholds, the AI tool had higher sensitivity, but lower specificity than the standard Brock score, suggesting use might complement clinician assessments of nodule risk.

## Highlights

• Artificial intelligence tool can identify and characterize nodules on screening CT.
• AI nodule risk prediction is similar to standard risk calculators.
• AI tool was more sensitive, but less specific than standard risk scores.
• Use of the AI tool may complement radiologist assessment of nodule risk.

## 1 Introduction

The increasing use of computed tomography (CT) scanning has led to an increase in the detection of pulmonary nodules, with an estimated 1.5 million nodules detected in the United States annually [1] Low dose chest CT (LDCT) screening for lung cancer also identifies a large number of nodules with 25% of baseline screens positive for a nodule. [2] Though the majority of CT detected nodules are benign, accurately identifying malignant nodules is essential for early lung cancer detection. The estimation of malignancy risk (pCA) is a crucial step in the evaluation of indeterminate pulmonary nodules (IPNs). [3]

Clinical risk prediction models have been developed to help clinicians estimate the pCA of CT-detected pulmonary nodules. Frequently utilized models include the Mayo model for incidental nodules and the Brock model for screen-detected nodules. [4], [5] These models incorporate both clinical and imaging characteristics to determine pCA, which in turn determines management strategies. Despite the reliance on radiologic nodule evaluation, there can be considerable variability between radiologists’ interpretation of nodule characteristics and their management recommendations. [6], [7], [8]

There has been growing interest in the development of artificial intelligence (AI) tools utilizing deep learning and convolutional neural networks to aid in the interpretation of CT images. [9], [10], [11] While the aim of many of these tools is nodule detection, other AI tools have been developed to characterize nodules and determine pCA. [9], [10], [11] Accurate pCA prediction could help streamline management decisions.

One such AI system (Qure.ai Technologies, Mumbai, India) has been developed to detect nodules on CT scan, and assess nodule characteristics including calcification, spiculation, and texture. [12] Incorporating the AI determined nodule characteristics with patient clinical information generates an automated malignancy risk prediction score utilizing the Brock model. The purpose of the study was to compare the performance of an AI generated Brock score with the standard Brock score to predict malignancy risk of screen-detected nodules.

## 2 Methods

### 2.1 Study design and participants

This was a retrospective case-control study utilizing a subset of data from the National Lung Cancer Screening Trial (NLST) previously granted for use to the Medical University of South Carolina (MUSC). [2] The study was approved by the MUSC institutional IRB (Pro00133896).

The NLST was a randomized controlled trial that compared the efficacy of lung cancer screening with LDCT to chest radiography. Detailed enrollment and methodology have been previously described. [2] Included patients were age 55–74 years, had smoking history of at least 30 pack-years, patients who were currently smoking or had quit within the past 15 years. Patients with a prior diagnosis of lung cancer, prior CT scan within 18 months of enrollment, or symptoms concerning for lung cancer were excluded. Patients were randomized to receive chest radiograph or LDCT at one-year intervals. The primary outcome was the reduction in lung cancer mortality.

The current study dataset was comprised of 299 lung cancer cases and 299 controls with benign nodules from the NLST as determined by pathology reports or radiographic stability during the NLST follow-up period. [2] Patients with benign and malignant pulmonary nodules detected on low dose CT scan (LDCT) were randomly selected from the CT screening group of the NLST. Pulmonary nodules were defined as non-calcified lesions ≥4 mm in size and included solid, part-solid and ground glass nodules. One hundred patients (50 cases, 50 controls) were randomly selected from the dataset for thresholding of the AI algorithm. The remaining 498 patients were analyzed by the AI algorithm masked to disease status to generate the AI Brock Score.

### 2.2 Standard and AI Brock score

The Brock score, which generates a probability of malignancy between 1 and 100, was calculated using the full-model with spiculation as described by McWilliams et al. [5] Variables required to calculate the standard Brock score were obtained from the NLST dataset which includes both clinical variables obtained at randomization and radiologic characteristics which were reported by board certified radiologists trained in image quality and standardized image interpretation. Clinical variables included age, sex, family history of lung cancer. Radiological variables included the diameter of lung nodules (long-axis diameter in mm), texture (solid, part-solid, ground glass), spiculation, location (right upper lobe, right middle lobe, right lower lobe, left upper lobe, left lower lobe), nodule count (total number of nodules present on the scan), and presence of emphysema.

For the AI Brock score, clinical variables were obtained from the NLST dataset. Since the AI tool does not determine the presence of emphysema, this was obtained from the NLST dataset. Nodule characteristics were obtained from the AI algorithm interpretation of CT images. Calcified nodules and nodules less than 4 mm (long-axis diameter) identified by the AI were removed from the analysis and nodule count for Brock score calculation. Additionally, if the AI detected more than 10 non-calcified nodules ≥ mm in diameter, the largest 10 nodules based on volume were included for the Brock score calculation and the nodule count was capped at a maximum of 10. Sensitivity analysis was performed at decreasing nodule count cap thresholds to assess changes in AUC, sensitivity, and specificity. If the AI did not detect a nodule or detected only calcified nodules or non-calcified nodule < 4 mm, a Brock score of zero was imputed for that case. For patients with more than one non-calcified nodule, the highest score was taken for the calculation of diagnostic performance metrics for both the standard and the AI Brock score.

### 2.3 Artificial intelligence tool

The AI tool, qCT (Qure.ai Technologies, Mumbai, India), is a fully automated deep-learning–based computer-aided detection (CAD) system designed to identify and characterize pulmonary nodules on chest CT scans. The algorithm was developed using an internal dataset comprising approximately 200,000 anonymized chest CT scans sourced from India, the Americas, and Europe. The dataset was divided in an 80:20 ratio, with 80% of scans used for model training and 20% reserved for internal validation.

The qCT framework consists of three major components: pre-processing, detection, and characterization. During pre-processing, input DICOM series are standardized into three-dimensional image volumes with uniform voxel spacing and intensity normalization. The detection stage employs a RetinaNet-based three-dimensional convolutional neural network (CNN) to localize regions likely to contain pulmonary nodules. Each potential nodule is assigned a confidence score between 0 and 1, representing the likelihood that the detected region corresponds to a true nodule.

Following detection, each candidate region undergoes segmentation and classification. A UNet3D-based CNN performs voxel-level segmentation to delineate the precise nodule boundaries, while a ResNet3D-based CNN classifies each nodule by key morphological features, including presence or absence of calcification, spiculated margins, and texture type (solid, part-solid, or ground-glass). The model also estimates quantitative parameters such as mean diameter and volume, and records lobe-level anatomical location.

To determine the optimal detection threshold for the study, 100 scans (50 cases and 50 controls) were randomly selected and reviewed in an unblinded fashion. The threshold defines the minimum confidence level required for a detected region to be considered a true positive nodule. All algorithms were pre-trained, locked, and internally validated prior to testing. Performance of the AI tool to detect and characterize nodules has been previously validated in an external dataset. [12]

### 2.4 Statistical methods

Descriptive statistics were used to characterize the cohort. Continuous variables were summarized using mean and standard deviation. Categorical variables were summarized using numbers and percentages. Chi-square for categorical and independent sample t test for continuous variable was used to test for differences in the characteristics between patients with lung cancer and benign nodules. The performance of the models was assessed using the area under the receiver operating curve (AUC). The 95% confidence interval (CI) of the AUC was constructed using DeLong’s method. The comparison between two AUCs was done using paired Delong’s test. The AUC with 95% CI was also calculated and compared across different sub-groups including sex, family history of lung cancer, emphysema, and number of nodules.

Sensitivity and specificity were calculated at fixed thresholds of ≥ 2, 5 and 10% of cancer risk. The 95% CI of the sensitivity and specificity was constructed using the modified Wilson’s score method. Overall percent agreement between standard Brock and the AI Brock at each of the above-mentioned thresholds were calculated along with Cohen’s kappa. Histogram of the standard Brock and AI Brock scores stratified by cancer diagnosis were generated to explore the distribution of scores. All statistical analyses were conducted using R (version 4.4.2, R Project for Statistical Computing).

## 3 Results

**Fig. 1:**
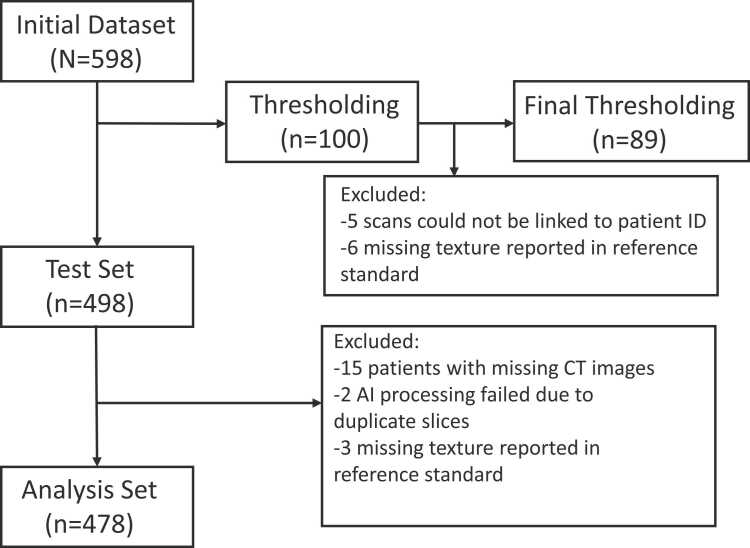
Study participant flow diagram. Flow of participants through the study from initial dataset. One hundred patients were randomly selected for thresholding of the artificial intelligence (AI) tool algorithm. Eleven patients were excluded due to failure to link scans to patient ID or missing data, leaving 89 patients for thresholding. Of the remaining 498 patients, 20 were excluded due to missing data or failed processing by the AI tool. The remaining 478 patients were used for the final analysis.

From the initial 598 patients (299 lung cancer cases and 299 benign nodule controls), 100 patients (50 cases, 50 controls) were randomly selected for thresholding of the AI algorithm. Of these 100 patients, 11 could not be processed by the AI software due to missing data, leaving 89 (45 cases, 44 controls) available for thresholding. Once optimal performance of the AI algorithm was determined, the detection threshold was set to 0.9. Of the remaining 498 patients, 20 were excluded due to missing data leaving 478 patients (239 cases, 239 controls) available for final analysis (Fig. 1). The mean age of patients was 63 (SD 5.5) years and 180 (38%) were females. Table 1 shows the distribution of clinical variables among those with lung cancer and benign nodules. Patients who developed cancer were slightly older (64 vs 62, p < 0.001) and had a higher prevalence of emphysema (56.8% vs 46.3%, p = 0.02).

**Table 1:** Distribution of demographic and clinical variables of 478 patients in the CT arm of the NLST.

**Variable**	**No lung cancer** **(n = 239)**	**Lung cancer** **(n = 239)**	**p-value**
Age (years)	61.95 (5.29)	63.79 (5.49)	< 0.001^a^
Sex			
Female	86 (47.78)	94 (52.22)	0.51^b^
Male	153 (51.34)	145 (48.66)	
Family history of lung cancer			
No	189 (51.78)	176 (48.22)	0.20^b^
Yes	50 (44.25)	63 (55.75)	
Emphysema			
No	85 (58.62)	60 (41.38)	0.02^b^
Yes	154 (46.25)	179 (53.75)	
Reported number of nodules*			
One	150 (52.82)	134 (47.18)	
2–4	82 (47.13)	92 (52.87)	0.19^a^
More than 4	7 (35.00)	13 (65.00)	

Reported as mean (SD) or n (%)

* Number reported in the reference standard sheet

^a^ Independent sample *t*-test

^b^χ^2^

After removing calcified lesions, lesions < 4 mm, and capping nodule count at 10, the AI tool identified 1638 nodules in 478 patients (3.4 per patient). The number of nodules present in the NLST reference standard sheet was 821 (average of 1.7 per patient). Zero was imputed as the AI Brock score for 15 (3%) patients, including six because all the nodules identified were characterized by AI as having calcification, five because the non-calcified nodule identified by the AI was < 4 mm, and four because the AI did not detect any nodule. In all cases, the NLST dataset reported at least one non-calcified nodule > 4 mm on the CT scan.

**Fig. 2:**
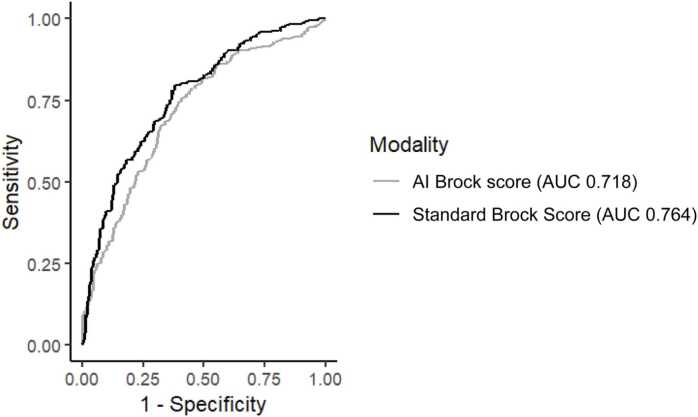
Receiver operating curve of standard Brock vs AI Brock score Receiver operating curves for the artificial intelligence (AI) Brock score (AUC=0.718) in gray and the standard Brock score (AUC=0.764) in black.

The AUC of the standard Brock’s score was 0.76 (95% CI 0.72 – 0.81) and the AUC of the AI Brock score was 0.72 (95% CI 0.672 – 0.764) (p = 0.053) (Fig. 2). Sensitivity analysis demonstrated no change in performance of the AI tool at decreasing nodule count caps. In subgroup analysis, the AUC for the standard Brock score was higher than the AUC for the AI Brock score in female patients (0.815, 95% CI 0.753 – 0.878 vs 0.742, 95% CI 0.670 – 0.814; p = 0.02), patients without emphysema (0.742, 95% CI 0.670 – 0.814 vs 0.684, 95% CI 0.594 – 0.774; p < 0.001), and in patients with only one nodule documented on the reference standard sheet (0.755, 95% CI 0.699 – 0.811 vs 0.683, 95% CI 0.620 – 0.746; p = 0.04), however there was no significant difference in the performance of the AI tool within subgroups (Table 2).

**Table 2:** Performance of Standard and AI Brock score among subgroups of patients (N = 478).

**Variable**	**Standard**	**AI**	p-value*
Sex			
Female (n = 180)	0.815 (0.753 – 0.878)	0.742 (0.670 – 0.814)	0.02
Male (n = 298)	0.735 (0.679 – 0.791)	0.701 (0.641 – 0.761)	0.31
Family H/o lung cancer			
No (n = 365)	0.739 (0.689 – 0.789)	0.699 (0.646 – 0.753)	0.17
Yes (n = 113)	0.836 (0.760 – 0.912)	0.767 (0.678 – 0.856)	0.10
Emphysema			
No (n = 145)	0.841 (0.775 – 0.906)	0.684 (0.594 – 0.774)	< 0.001
Yes (n = 333)	0.726 (0.672 – 0.780)	0.723 (0.669 – 0.777)	0.92
Number of nodules			
One (n = 284)	0.755 (0.699 – 0.811)	0.683 (0.620 – 0.746)	0.04
2–4 (n = 174)	0.786 (0.719 – 0.853)	0.773 (0.704 – 0.841)	0.69
Greater than 4 (n = 20)	0.714 (0.446 – 0.983)	0.736 (0.494 – 0.978)	0.88

Reported as AUC (95% Confidence Interval)

*Paired Delong’s test

AI: Artificial Intelligence, h/o: history of

**Fig. 3:**
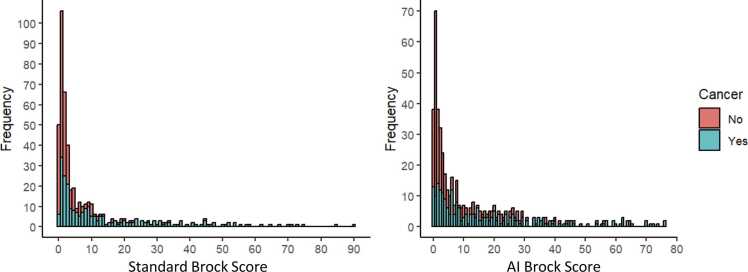
Histogram of standard and AI Brock score by cancer diagnosis Distribution of Brock scores both for the AI tool and the standard Brock score for patients with lung cancer (blue) and for patients without cancer (red).

Table 3 shows the sensitivity and specificity of AI and standard Brock score at fixed thresholds of ≥2, 5, and 10% pCA and the overall agreement and the strength of the agreement between the standard and AI Brock. At all selected thresholds, the standard Brock score had a higher specificity, and the AI Brock had a higher sensitivity. The agreement between the standard and the AI Brock score at these thresholds ranged from 0.37 to 0.46, indicating fair to moderate agreement. Fig. 3 shows the histogram of both scores stratified by cancer status. For patients with cancer, the mean standard Brock score was 14.4% (SD 17.55) while the AI Brock was 18.78% (SD 19.56). For those with benign nodules the mean standard Brock was 4.30% (SD 9.30) and the AI Brock was 7.43% (SD 10.90).

**Table 3:** Sensitivity and specificity of Standard and AI Brock score at fixed thresholds of pCA.

**Threshold**	**Standard**		**AI**		**Agreement**	
	***Sensitivity*** ***(n = 239)***	***Specificity*** ***(n = 239)***	***Sensitivity*** ***(n = 239)***	***Specificity*** ***(n = 239)***	**Agreement (%)**	**kappa**
> 2%	76.99 (71.25 – 81.87)	63.18 (56.90 – 69.04)	83.26 (78.01 – 87.46)	45.61 (39.41– 51.94)	70.08	0.37
> 5%	56.90 (50.57 – 63.02)	82.01 (76.64 – 86.36)	68.62 (62.48 – 74.17)	65.27 (59.04 – 71.02)	72.80	0.46
> 10%	39.75 (33.75 – 46.07)	90.38 (85.97 – 93.50)	53.14 (46.81 – 59.36)	77.41 (71.69 – 82.25)	75.94	0.45

Reported as percentage (95% confidence interval)

AI: Artificial Intelligence, pCA: probability of malignancy

At a ≥5% risk threshold, the standard Brock score accurately predicted malignancy in 136/239 (56.9%) of cases, AI Brock score in 164/239 (68.5%) of cases and both in 121/239 (50.6%) of cases. The AI Brock score misclassified 15 of those correctly predicted by the standard Brock score and the standard Brock score misclassified 43 of those correctly predicted by the AI Brock score. Both misclassified 60 cancer cases. Among the patients with benign nodules, the standard Brock score accurately predicted 196/239 (82%) of controls, AI Brock score predicted 156/239 (65%) and both predicted 140/239 (58.6%). The AI Brock score misclassified 56 of those correctly predicted by the standard Brock score and the standard Brock score misclassified 16 of those correctly predicted by AI Brock score. Both misclassified in 27 benign controls.

## 4 Discussion

This study evaluated the performance of an AI generated Brock score to predict malignancy in screen-detected pulmonary nodules. The performance of the AI Brock score was lower than the standard Brock and the results approached statistical significance. The AI tool identified many more nodules than were initially reported in the NLST dataset. The higher nodule detection rate likely contributed to the increased sensitivity but decreased specificity of the AI Brock score compared with the standard Brock score across all risk thresholds.

Clinical risk prediction models were developed to assess the risk of malignancy (pCA) for nodules identified on imaging. Although these models often include clinical characteristics such as age, history of smoking, or family history of lung cancer, nodule characteristics contribute a large proportion of cancer risk. [4], [5] The Brock model, developed in a high-risk screening population with a low prevalence of malignancy, predicts malignancy based on four clinical risk factors and five nodule characteristics. Consider for example a 70-year-old female with emphysema and a family history of lung cancer. If she were to develop a single, non-spiculated 8 mm ground glass nodule, not in the upper lung zone, the model would estimate the risk of malignancy to be 6%. However, were she to develop a single 13 mm, spiculated, part solid nodule in an upper lobe, the model-based estimate of risk of malignancy would increase to 62% (Table 4). [5] This change in risk highlights the importance of accurately identifying and characterizing nodules on CT images.

**Table 4:** Risk of malignancy based on clinical and nodule characteristics.

Predictors	Patient 1	Patient 2	Patient 3
Clinical Risk Factors	Age: 70 Sex: Female Family History: yes Emphysema: yes	Age: 70 Sex: Female Family History: Yes Emphysema Yes	Age: 60 Sex: male Family History: no Emphysema: no
Nodule Risk Factors	Size: 8 mm Type: ground glass Upper lung: no Count: 1 Spiculation: no	Size: 13 mm Type: part-solid Upper lung: yes Count: 1 Spiculation: yes	Size: 13 mm Type: part-solid Upper lung: yes Count: 1 Spiculation: yes
Brock calculated malignancy risk	6%	62%	26%

Assessment of pCA requires accurate radiological assessment and reporting of nodule characteristics. However, even highly trained radiologists are not immune to errors. There can be considerable variability in the number of nodules identified amongst experienced radiologists. [13], [14] Radiologists may also succumb to “satisfaction of search” if there is an obvious abnormality on the scan and fail to identify more subtle abnormalities such as small nodules. [15] Since higher nodule count reduces the probability of malignancy in the Brock model, identification of all nodules on a scan is essential. Several studies have demonstrated that computer-aided detection systems could improve radiologists’ detection rate of pulmonary nodules. [9], [10], [16] In the current study, the AI tool identified approximately twice as many nodules as were reported by radiologists in the NLST. It is possible that the AI system identified nodules that were missed by radiologists, however it is also possible that the AI tool incorrectly identified findings as nodules rather than other findings such as blood vessels or intrapulmonary lymph nodes. As the present study did not have a process for adjudicating discrepancies between the radiology report and AI tool it is unclear which count was more accurate. Though decreasing the nodule count cap did not affect the overall performance of the AI tool, the higher nodule detection rate could have contributed to the lower specificity of the AI Brock model by assigning risk scores to benign findings that were not flagged by radiologists. However, it is also possible that the higher nodule detection rate contributed to the increased sensitivity of the AI tool.

In addition to nodule count, other nodule characteristics such as size, density, and the presence of spiculation are prone to reader variability. [17], [18] One study showed that size measurements for small pulmonary lesions can vary as much as 20% even among the same reader for scans taken less than 15 min apart. [17] Other studies have proposed that automated volumetric measurement systems can improve on manual measurements. [19] Analysis of subsolid nodules, both ground glass and part-solid nodules, can be particularly challenging due to variability in interpretation of nodule density and size of the solid component. [7], [18] Discrepancies in radiologists’ identification and characterization of nodules can lead to variability in management recommendations. [6], [8], [20] Although the AI tool evaluated in this study did not outperform the standard Brock score, it could serve as a helpful adjunct for radiologist interpretation of nodules, either by identifying the most suspicious lesion for closer inspection, identifying nodules that may have been missed by the radiologist, or assisting with nodule characterization. However, it is also possible that inaccurate flagging of nodules by the AI tool could lead to incorrect management recommendations, thus leading to patient harm. [21] Understanding the benefits or harms of the AI tool when incorporated into a clinical workflow requires further evaluation.

Other AI tools have been developed to aid in nodule detection and risk characterization. Liu et al. developed a deep learning convolutional neural network to detect pulmonary nodules on CT images which improved radiologist nodule detection sensitivity and decreased reading times. [9] Murchison et al. validated a deep learning computer aided detection system to detect and characterize pulmonary lesions and demonstrated improvement in sensitivity of nodule detection, however at a higher false positive rate. [10] Other AI systems have moved beyond nodule identification to assign unique malignancy risk scores, separate from traditional clinical risk prediction models. [11], [22] The AI tool evaluated in this study serves as a hybrid of these two approaches by identifying and characterizing nodules and providing a malignancy risk score based on a known clinical risk prediction model. However, the AI tool requires input of clinical variables, such as age, sex, and family history of lung cancer which may not be readily available to radiologists interpreting images and could impede workflow. For example, if a radiologist were required to review the patient’s chart to ascertain family history of lung cancer and manually enter the data into the AI tool, this may negate the potential time saved by the tool’s automatic detection and nodule characterization. Another limitation of the AI tool is the removal of all nodules with calcification from consideration as malignant nodules, which led to misclassification of 6 malignant nodules. This is because the AI tool classifies calcification as a binary yes/no and cannot distinguish between benign and suspicious calcification patterns. [23] Further work is needed to refine the algorithm for the AI tool to detect malignant lesions with calcifications. Furthermore, though the Brock score was developed to assess risk in screen detected nodules, it was not validated on follow-up scans that may demonstrate growth or stability and its current clinical use is limited as radiologists in the United States typically use LungRADs to guide nodule management. [24]

The study has several limitations. First, this is a retrospective study with a relatively small sample size, however it offers a large number of lung cancer cases for assessment which would generally require a much larger cohort due to the overall low incidence of lung cancer in screening populations. [2] Second, there was not a method for adjudicating discrepancies between the AI software and the initial NLST dataset, although given that the original data was collected as part of a randomized clinical trial and had up to 7 years of follow-up time, the NLST data were utilized as the reference standard for this study. Third, the NLST data linked cancer diagnosis at the patient level rather than the nodule level, which could have led to misclassification bias for patients with multiple nodules. Further evaluation of the tool in a larger prospective cohort would be valuable.

In conclusion, the AI tool evaluated in this study achieved a lower performance than the standard Brock score, though the results did not quite meet statistical significance. Across all risk thresholds, the AI tool missed fewer cancers at the expense of a higher rate of misclassification of benign nodules. Further development of the model should evaluate the probability of cancer in a pulmonary nodule without requiring input of clinical variables not routinely available in radiology requests. Future work is needed to assess the impact of the AI tool on clinical workflow and patient outcomes.

## CRediT authorship contribution statement

**Silvestri Gerard:** Writing – review & editing, Supervision, Conceptualization. **Deepak Adarsh:** Methodology, Formal analysis. **Vikash Challa:** Software, Formal analysis. **Saigopal Sathyamurthy:** Software, Formal analysis. **Michael Balassone:** Resources, Project administration, Data curation. **Long Kathryn:** Writing – original draft, Methodology, Conceptualization.

## Funding

Qure.ai Technologies, Mumbai, India

KJL was supported by T32 training grant 10.13039/100000002NIH-T21-HL144470 at time of this work.

## Declaration of Competing Interest

SS, VC, and DA disclose employment with Qure.ai Technologies

KJL, GAS and MB have no relevant disclosures

## Data Availability

Data generated or analyzed during the study are available from the corresponding author by request.
